# Complete mitochondrial genome and phylogenetic analysis of the marine red alga *Polyopes affinis* (Rhodophyta: Halymeniales)

**DOI:** 10.1080/23802359.2022.2101399

**Published:** 2022-07-28

**Authors:** Maheshkumar Prakash Patil, Jong-Oh Kim, Kyunghoi Kim, Young-Ryun Kim, Seokjin Yoon

**Affiliations:** a Industry-University Cooperation Foundation, Pukyong National University, Busan, Republic of Korea; b Department of Microbiology, Pukyong National University, Busan, Republic of Korea; c School of Marine and Fisheries Life Science, Pukyong National University, Busan, Republic of Korea; d Department of Ocean Engineering, Pukyong National University, Busan, Republic of Korea; e Marine Eco-Technology Institute, Busan, Republic of Korea; f Dokdo Fisheries Research Center, National Institute of Fisheries Science, Pohang, Republic of Korea

**Keywords:** Halymeniales, Rhodophyta, mitochondrial genome, phylogenetic analysis, *Polyopes affinis*

## Abstract

*Polyopes affinis* ((Harvey) Kawaguchi & Wang, 2002) is a red alga in the order Halymeniales of the phylum Rhodophyta. The entire mitogenome of *P. affinis* was sequenced and compared to related Halymeniales species. The entire circular-mitogenome is 25,988 bp long, has 27.59% GC content, and comprises 25 protein-coding genes (CDS), 23 transfer RNA (tRNA) genes, and three ribosomal RNA (rRNA) genes. In terms of gene synteny and tRNA composition, the *P. affinis* mitogenome differs significantly from that of *P. lancifolius*. Phylogenetic analysis shows *P. affinis* mitogenome in a branch sister to *P. lancifolius*, indicating a close relationship with other Halymeniales species.

*Polyopes affinis* is a red alga from the phylum Rhodophyta's order Halymeniales. This species is abundantly found along the South Korean coast (East and South) and Japan, and it is widely used as a food in South Korea, Japan, and China because of its nutritional properties and health benefits (Ha et al. 2022). The mitochondrial genome (mtDNA) is important for biogeographic and population genetic studies, as well as molecular evolution investigations (Kim et al. 2012). There are four species in the Halymeniales that have complete mtDNA sequences. The complete mtDNA of *P. affinis*, the second species in the genus *Polyopes*, was sequenced and described in this work.

The specimen which was collected by a diver in the East Sea, Republic of Korea (37°06′41.5″N 129°22′45.6″E) with voucher specimen number PU-T01-S-MA-01 was deposited at the Ecological Restoration Group, Marine Eco-Technology Institute, Busan, Republic of Korea (Young-Ryun Kim, yykim@marine-eco.co.kr). The genomic DNA was extracted using DNeasy Blood and tissue kit (Qiagen, Hilden, Germany) following the manufacturer’s instructions. A DNA library was prepared using TruSeq Nano DNA Kit and sequenced on the Illumina platform (Illumina, San Diego, CA). After completing mtDNA *de novo* assembly with the SPAdes 3.13.0 (Bankevich et al. 2012), the MFannot tool (https://megasun.bch.umontreal.ca/cgi-bin/mfannot/) was used to annotate protein-coding genes (CDS), transfer RNA (tRNA) genes, and ribosomal RNA (rRNA) genes. A maximum-likelihood phylogenetic tree was constructed using the MEGA11 version 11.0.8 software (Tamura et al. 2021) and 1000 bootstrap alignments based on complete mtDNA sequences obtained from the NCBI database (https://www.ncbi.nlm.nih.gov/).

The complete circular mtDNA (GenBank accession no.: OM960741) of *P. affinis* was 25,988 bp in length with 27.59% GC content, which is lower than *P. lancifolius* (MW292567; Kim et al. 2021). The overall base composition was 37.85% for A (9837 bp), 34.55% for T (8979 bp), 14.25% for G (3704 bp), and 13.34% for C (3468 bp). It contains a total of 51 genes, including three *rRNA* genes, 23 *tRNA* genes, and 25 CDS genes. The CDS genes include seven for NADH dehydrogenase complex (nad), five for ribosomal proteins (rsp), four for ATPase subunits (atp), three for cytochrome oxidase subunits (cox), three for sdh, one for cob, one for tatC, and one for hypothetical protein. The genomic difference between the two *Polyopes* species is that *P. lancifolius* (Kim et al. 2021) has fewer rRNA (2), and CDS (24) than *P. affinis*. While there are differences amongst Halymeniales species, the mtDNA of *P. affinis* (OM960741) and *P. lancifolius* (MW292567) lacks an intron in cox1, which is found in *Grateloupia taiwanensis* (KM999231), *G. filicina* (KG598532), and *G. angusta* (KC875853).

A maximum-likelihood phylogenetic tree (Figure 1) was constructed using six mtDNA within the order Halymeniales. *Sebdenia flabellata* (KJ398164) and *Rhodymenia pseudopalmata* (KC875852) were used as outgroups. The results are consistent with previous findings, and *P. affinis* was found on a branch sister to *P. lancifolius* in the best-scoring tree, revealing a close relationship with other Halymeniales species. This complete mtDNA analysis of *P. affinis* will improve our understanding of the evolutionary process of Rhodophyta species.

**Figure 1.**
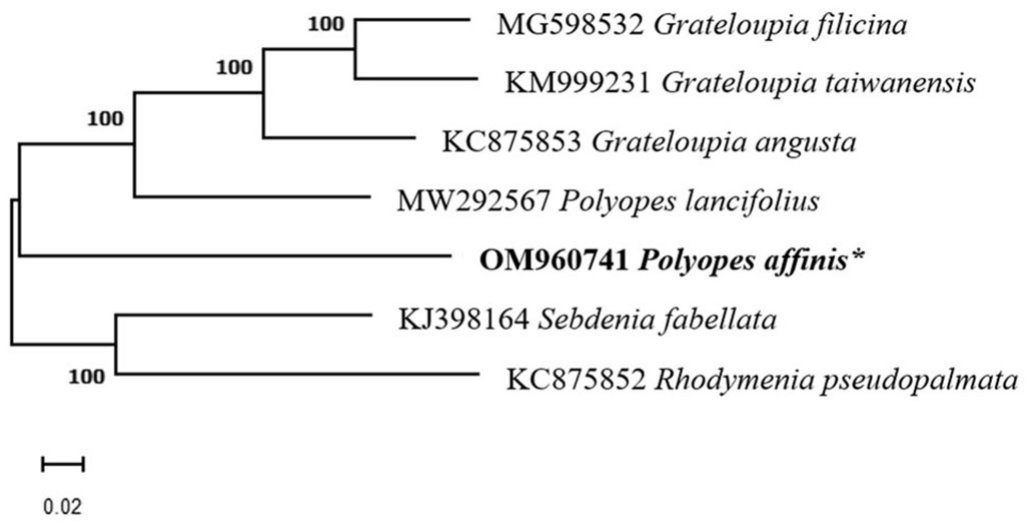
Phylogenetic tree (maximum likelihood) of the order Halymeniales based on seven complete mitogenome sequences, with *Sebdenia flabellata* and *Rhodymenia pseudopalmata* used as outgroups (bootstrap values based on 1000 replicates). Asterisks following species names indicate the newly determined mitochondrial genome.

## Data Availability

The genome sequence data that support the findings of this study are openly available in GenBank of NCBI (https://www.ncbi.nlm.nih.gov/) under the accession number OM960741 (https://www.ncbi.nlm.nih.gov/nuccore/OM960741). The associated BioProject, BioSample, and SRA numbers are PRJNA825647 (https://www.ncbi.nlm.nih.gov/bioproject/PRJNA825647/), SAMN27531872 (https://www.ncbi.nlm.nih.gov/biosample/SAMN27531872/), and SRR18728299 (https://www.ncbi.nlm.nih.gov/sra/SRR18728299/), respectively.
